# Parental views of children’s physical activity: a qualitative study with parents from multi-ethnic backgrounds living in England

**DOI:** 10.1186/s12889-015-2351-8

**Published:** 2015-10-02

**Authors:** Joanne Trigwell, Rebecca Catherine Murphy, Nigel Timothy Cable, Gareth Stratton, Paula Mary Watson

**Affiliations:** Institute for Health and Wellbeing, Leeds Beckett University, Leeds, UK; Physical Activity Exchange, Research Institute for Sport and Exercise Sciences, Liverpool John Moores University, Liverpool, UK; Aspire Academy, Doha, Qatar; College of Engineering, Swansea University, Swansea, UK

**Keywords:** Children, Ethnicity, Parents, Physical activity, Qualitative

## Abstract

**Background:**

Guidelines recommend children and young people participate in at least 60 min of physical activity (PA) every day, however, findings from UK studies show PA levels of children vary across ethnic groups. Since parents play an instrumental role in determining children’s PA levels, this article aims to explore parental views of children’s PA in a multi-ethnic sample living in a large city in the North-West of England.

**Methods:**

Six single-ethnic focus groups were conducted with 36 parents of school-aged children (4 to 16 years) with a predominantly low socio-economic status (SES). Parents self-identified their ethnic background as Asian Bangladeshi (*n* = 5), Black African (*n* = 4), Black Somali (*n* = 7), Chinese (*n* = 6), White British (*n* = 8) and Yemeni (*n* = 6). Focus group topics included understanding of PA, awareness of PA guidelines, knowledge of benefits associated with PA and perceived influences on PA in childhood. Data were analysed thematically using QSR NVivo 9.0.

**Results:**

Parents from all ethnic groups valued PA and were aware of its benefits, however they lacked awareness of PA recommendations, perceived school to be the main provider for children’s PA, and reported challenges in motivating children to be active. At the environmental level, barriers to PA included safety concerns, adverse weather, lack of resources and lack of access. Additional barriers were noted for ethnic groups from cultures that prioritised educational attainment over PA (Asian Bangladeshi, Chinese, Yemeni) and with a Muslim faith (Asian Bangladeshi, Black Somali, Yemeni), who reported a lack of culturally appropriate PA opportunities for girls.

**Conclusion:**

Parents from multi-ethnic groups lacked awareness of children’s PA recommendations and faced barriers to promoting children’s PA out of school, with certain ethnic groups facing additional barriers due to cultural and religious factors. It is recommended children’s PA interventions address influences at all socio-ecological levels, and account for differences between ethnic groups.

## Background

A lack of regular physical activity (PA) is associated with an increased risk of developing obesity, as well as other metabolic and cardiovascular disease [1]. Guidelines recommend children and young people engage in at least 60 min of moderate-to-vigorous physical activity (MVPA) per day and undertake vigorous intensity activities at least three days per week [2]. Due to the health consequences associated with sedentary behaviours, including increased risk of obesity independent of moderate to vigorous PA levels [3, 4], PA guidelines also recommend reducing total sedentary time and breaking up extended periods of sitting [2]. Recent data from the Health Survey for England showed only 21 % of boys and 16 % of girls meet daily PA recommendations, and average weekday sedentary time (excluding school) is 3.3 h for boys and 3.2 h for girls [5]. Furthermore, findings from UK studies show PA levels of children vary across ethnic groups, with Black and Asian children generally less active [6, 7] and more sedentary than their White counterparts [8].

A socio-ecological approach to health promotion suggests factors which influence health behaviours occur within a multi-layered context [9]. Influences on children’s PA behaviours range from individual (e.g., children’s preferences) to interpersonal (e.g., parental support), organisational (e.g., school PA provision) and environmental (e.g., local PA opportunities) levels and act either independently or synergistically to impact on children’s behaviour [10, 11]. At the interpersonal level, parents play an instrumental role in determining children’s PA levels through their attitudes, parenting practices and own PA behaviours [12, 13]. Cultural factors may influence how children’s PA is perceived and supported by parents of different ethnic groups [14]. Qualitative evidence suggests cultural attitudes can act as a barrier to PA, such as over-protection in Chinese parents [11], an emphasis on educational attainment in Middle-Eastern parents [11], and cultural expectations of girls from Black Somali parents (e.g., certain sports deemed unsuitable for girls, requirements for female-only physical activities that allow cultural dress ) [15]. Furthermore Bangladeshi and Somali teenagers living in Wales have reported a lack of support from their families to do PA, which boys felt was because of injury worries [16]. The UK Department of Health [17] provided insight into differences in parental attitudes between ethnic minority groups living in England, showing some groups tended to prioritise educational commitments over organised sport (e.g., Bangladeshi, Pakistani and Black African), whereas other groups allowed their children more freedom to engage in physical activities after school (e.g., Gujarati Hindu, Punjabi Sikh, Black Caribbean).

At the environmental level, living in Western countries may act as both a negative and positive influence on PA for ethnic minority children. For example, parents and children of Somali origin living in the US have reported barriers to PA in winter months such as lack of transport or lack of resources to buy appropriate winter clothing [15]. However cultural factors can also facilitate PA, as Middle-Eastern parents living in Australia reported how having extended family living in the same street reduced their PA-related safety concerns [11]. In this regard, it is possible the demographic make-up of a city might influence the PA of ethnic minority children. For example, children from ethnic minority groups living in predominantly White-British cities may have different experiences of PA when compared with children living in cities of high ethnic diversity (where non-White British groups represent a substantial proportion of the population). There may be positive influences through being part of a Western culture in which PA is a public health priority but there may also be negative factors, for example less consideration of the needs of Muslim girls in school and fewer appropriate PA opportunities in the local community.

The health inequalities faced by ethnic minority groups may be rooted in social, cultural and environmental factors [18] and research is needed to understand the influence of these factors on health behaviours such as PA. For sustainable and effective health promotion, it is important interventions are informed by the voices of the communities they seek to help (e.g., low socio-economic groups, ethnic minority groups) [19, 20]. Therefore qualitative methods are required to explore the views of ethnic minority groups to ensure health promotion efforts reflect recipients’ knowledge, attitudes and perceptions about PA behaviours. Previous research into parental views of children’s PA in England [17] has focussed on cities with a high ethnic diversity and it is not known how the findings transfer to areas where ethnic minority groups represent only a small percentage of the population. This qualitative study therefore explores parental views of children’s PA (knowledge, attitudes and perceived influences) among a multi-ethnic sample living in Liverpool, a deprived local authority in the North West of England [21] with a low proportion of ethnic minority residents in comparison to the national average [22]. A focus group methodology is used to explore similarities and differences in parental perspectives across six different ethnic groups (Asian Bangladeshi, Black African, Black Somali, Chinese, White British and Yemeni).

## Methods

This study was part of a larger research project designed to improve the cultural relevance of family-based childhood obesity treatment for ethnic minority groups [23]. Recognising the value of qualitative methodology in formative research [24], a focus group design was employed to acquire the necessary context and depth to interpret individuals’ experiences. The study adhered to the RATS guidelines for qualitative studies [25] to ensure the relevance of study question, appropriateness of methods, transparency of procedures and soundness of interpretations.

### Sample and recruitment

Parents of children aged 4 to 16 years who self-identified their ethnic background as Asian Bangladeshi, Black African, Black Somali, Chinese, White British and Yemeni were eligible to take part. Participants were recruited from a sample of parents who had previously returned a questionnaire investigating their perceptions of overweight in childhood [26]; thus, ethnic groups were selected based on this earlier research. Purposive sampling techniques were employed to select parents who participated in the previous study based on their proximity to the location of the focus group venue. Letters were sent to parents who had consented to further contact from the research team and were followed up with telephone calls. Where participant numbers were low for particular ethnicities (Asian Bangladeshi, Black Somali, Chinese and Yemeni groups), parents were also recruited through schools and community centres by teaching staff and community workers. As a result, focus groups were conducted with a mix of parents who did and did not have established relationships with other participants prior to the study.

Six single-ethnicity focus groups were conducted, including 36 parents in total (5 Asian Bangladeshi, 4 Black African, 7 Black Somali, 6 Chinese, 8 White British, 6 Yemeni). Social demographics were collected from parents via a short self-report questionnaire (see Table 1). Although parents of both genders were invited to take part, the groups were predominantly female, with only the Black African and Chinese groups including both females and males. Age ranged between 29 and 54 years and 29 participants (80.6 %) lived within the 10 % most deprived areas in England [21]. With the exception of the White British group, only six participants were born in the UK (1/5 in the Asian Bangladeshi group, 1/6 in the Chinese group and 4/6 in the Yemeni group). For those not born in the UK, time living in the UK ranged from ≤5 years to ≥26 years. The participants in three of the groups were of Muslim religion (Asian Bangladeshi, Black Somali, Yemeni), one group were Christian (Black African) and two groups contained a mixture of religious backgrounds (Chinese, White British).

**Table 1:** Sample demographics

	Asian Bangladeshi	Black African	Black Somali	Chinese	White British	Yemeni
	*(n* = *5*)	(*n* = 4)	(*n* = 7)	(*n* = 6)	(*n* = 8)	(*n* = 6)
Gender	Female *n* = 5	Female *n* = 2	Female *n* = 7	Female *n* = 5	Female *n* = 8	Female *n* = 6
		Male *n* = 2				
				Male *n* = 1		
Age	Mean = 35.8 years (range, 30–40 years)	Mean = 39.0 years (range, 33–40 years)	Mean = 44.6 years (range, 33–54 years)	Mean = 36.5 years (range, 31–44 years)	Mean = 38.3 years (range, 29–52 years)	Mean = 35.2 years (range, 29–42 years)
				Missing *n* = 2	Missing *n* = 2	Missing *n* = 1
Religion	Muslim *n* = 5	Christian *n* = 4	Muslim *n* = 7	Buddhist *n* = 2	Christian *n* = 6	Muslim *n* = 6
				No religion *n* = 2	No religion *n* = 1	
				Missing *n* = 2	Missing *n* = 1	
Place of birth	UK *n* = 1	Africa *n* = 4	Somalia *n* = 7	UK *n* = 1	UK *n* = 8	UK *n* = 4
	Bangladesh *n* = 4			China *n* = 5		Yemen *n* = 2
Years lived in the UK (if not born in the UK)	0–5 years *n* = 1	0–5 years *n* = 2	6–10 years *n* = 1	0–5 years *n* = 1	n/a	6–10 years *n* = 1
			11–15 years *n* = 3			
	16–20 years *n* = 1		16–20 years *n* = 1	6–10 years *n* = 3		
		11–15 years *n* = 2				
			21–25 years *n* = 1			
	21–25 years *n* = 2		Missing *n* = 1	26+ years *n* = 1		
						Unknown *n* = 1
Main language spoken (if not born in the UK)	Bengali *n* = 4	English *n* = 4	Somali *n* = 7	Mandarin *n* = 5	n/a	Arabic *n* = 2
How well English is spoken (if English not first language)	Very well *n* = 1	n/a	Well *n* = 2	Not well *n* = 2	n/a	Well *n* = 2
				Not at all well *n* = 2		
	Well *n* = 3		Not at all well *n* = 5	Missing *n* = 1		
Index of Multiple Deprivation (IMD)*	Level 1 *n* = 2	Level 1 *n* = 4	Level 1 *n* = 7	Level 1 *n* = 3	Level 1 *n* = 8	Level 1 *n* = 5
	Level 2 *n* = 2			Level 2 *n* = 1		Level 2 *n* = 1
	Level 7 *n* = 1			Missing *n* = 2		

*IMD is a proxy for socio-economic status. IMD scores are a composite of seven domains of deprivation (income, employment, education, health, crime, access to services, and living environment). Level 1 = ranked within 10 % most deprived neighbourhoods in England; Level 2 = ranked within 20 % most deprived neighbourhoods in England; Level 7 = ranked within the 40 % least deprived neighbourhoods in England [21]

### Procedure

Focus groups were conducted between February and June 2010. All focus groups were held in local primary schools and community centres after school hours or at weekends based on the preferences of participants. Free crèche facilities were provided. A White British researcher (JT) facilitated all groups conducted in English (Asian Bangladeshi, Black African, White British and Yemeni groups). The Black Somali group was facilitated in Somali by a researcher from a Somali background (with JT present) and the Chinese group was facilitated in Mandarin by a researcher from a Chinese background (with JT present). In the Asian Bangladeshi and Yemeni groups, an interpreter provided bi-lingual support for JT as required, though the assistance was minimal due to a good level of English in these groups. All facilitators and interpreters were female, had knowledge of the research project and were experienced in focus group delivery.

Focus groups were conducted using semi-structured topic guides. The semi-structured guides were designed to permit participants to respond freely but also ensured significant topics were covered in detail and allowed for a degree of comparability across the transcripts [27]. The topic guides underwent extensive revision prior to the data collection phase to ensure acceptability of questions across ethnic groups, and input from ethnic minority and bi-lingual community workers, parents, and researchers were instrumental in this. As recommended, questions used in the topic guide were also discussed for semantic and conceptual equivalence in meaning when translated into the target languages [28]. During focus groups, parents were asked about their perceptions of body image, healthy eating and PA during childhood. This article focusses on data related to PA only. Key areas for discussion included *understanding of PA*, *awareness of PA guidelines*, *knowledge of benefits associated to participating in PA* and *perceived influences on PA in childhood*. Throughout the focus groups, participants were encouraged to air their honest views, even if these were in disagreement with others. Facilitators were trained to use active listening and paraphrasing techniques to help participants feel at ease and check their understanding of participant meaning [29]. Each focus group lasted approximately 1 h.

Ethical approval was granted from the local NHS Research Ethics Committee [ref: 09/H1017/86] and informed written consent obtained from all participants. Participants were given a £10 voucher as a thank you for their time.

### Data analysis

Focus group discussions were audio recorded with permission from participants and transcribed verbatim for analysis. This enabled a comprehensive inspection [30] and avoided inaccurate recollection of conversations [27]. During this process care was taken to ensure the anonymity of all data. Where focus groups were not facilitated in English, recordings were transcribed in the original language and later translated through a one-way method into English for analysis.

Transcripts were imported into QSR NVivo 9.0 and analysed thematically [31] by JT, with frequent debriefing sessions with PW and RM to discuss, debate and refine emerging themes. Transcripts, audio files and field notes from each group were collated and data were coded; this process involved reading and re-reading text and assigning broad thematic codes, some of which were pre-defined, based on topics covered in the group schedule and the socio-ecological model. Therefore, a combination of inductive analysis and deductive techniques were used to generate codes. All data was coded under at least one broad heading, but notably, some data were coded several times. Subsequently, broad codes were collapsed into higher and lower order themes and descriptive and interpretive summaries were written based on recursive engagement with the data.

## Results

In terms of *knowledge and understanding of PA* and the majority of themes related to *perceived influences on PA*, there were no observable differences between ethnic groups. Some groups did however cite additional religious and cultural barriers to children’s PA. The cross-group themes will be presented first, followed by influences specific to certain ethnic groups.

### Knowledge and understanding of PA

Parents recognised various benefits to being physically active in childhood; benefits related to health and physical development (the prevention of disease, weight management, body development); psycho-social state (increased sense of happiness, improved well-being, mentality, fit-in with peers); skill acquisition; and establishing healthy lifestyle habits for the future (see Table 2).

**Table 2:** Benefits associated to PA in childhood

	Example quote
Health and physical development (the prevention of disease, weight management, body development)	‘…*it [PA] leads the child to be healthy’. (Mother 1, Black Somali group)*
	*‘I think it’s important because it builds up their body, its builds up a stronger body’. (Father 2, Black African group)*
Psycho-social state (increased sense of happiness, improved well-being, mentality, fit-in with peers)	*‘They feel happy, they feel happy [when they do exercise] and when they don’t do exercise it makes… they don’t feel happy’. (Mother 2, Black African group)*
	*‘…I always want him to participate in PA and that's why I keep coming back to physical activities. I want him to participate in school as fully to fit in, not to just fit in, to, to make friends, in these groups that they have’. (Mother 4, Yemeni group)*
Skill acquisition	*‘We just hope our children can fully develop their skills, I will let him try everything’. (Mother 4, Chinese group)*
Establishing healthy lifestyle habits for the future	*‘He’s doing karate at the moment, which I know loads of people, the kids, the rest of the kids are doing as well but I just feel that if he doesn’t do that when he’s young he’ll never to that when he’s older. If he hasn’t got the mentality to keep fit when he’s younger he will never have it when he grows older’. (Mother 3, Yemeni group)*

PA was defined as anything from *“everything that actually involves moving your body”* to “*like real exercise”*. Confusion was exhibited regarding the intensity of activity necessary to gain the associated health outcomes. Across groups, some parents recognised that a particular level of intensity within an activity is required to gain the greatest health benefits. These parents referred to *“proper”* PA, which included *“running around”* or *“like a football session*, *karate sessions*, *go swimming”*. Other parents considered any movement as important.

*Mother 3: … as long as they’re doing something you know kicking the ball or out on their bike*

*Mother 7: Yeah, as long as they’re moving*

*(White British group)*

Generally, parents were unaware of PA recommendations for children and estimated the maximum amount of time children should spend screen watching to range between 30 min and three hours a day. In all ethnic groups parents considered the PA children did at school, during PE classes, at break time and/ or when walking to and from school to fulfil the recommendation on school days.

*‘It’s OK because already they do this in school, PE, running, PE, they’re doing this in school’. (Mother 3, Asian Bangladeshi group)*

### Perceived influences on PA in childhood

#### Intrapersonal influences

Parents perceived intrapersonal influences on participation in PA to relate to children’s health and overweight status as well as intrinsic motivations. Children who were considered to be overweight or to have health problems were viewed as less able or motivated to participate in PA.

*‘For example if a child is already fat, that means he will have less interest in sport’. (Mother 2, Chinese group)*

Moreover, parents recognised a lack of motivation, often associated to preferences for sedentary behaviours, to have a negative impact on children’s activity levels.

*‘Or when you see them being very lazy, so you’ll say “oh come on shall we walk”, for example to the* [local supermarket]*, and they’ll go “oh no I can’t be bothered or I just want to sit here and watch this programme”, and they’ve got programme after programme to watch’. (Mother 1, Yemeni group)*

#### Interpersonal influences

The school environment was considered primarily responsible for children’s PA levels (through structured and unstructured opportunities). However, parents also deemed themselves both facilitators and barriers to PA in childhood. Parental encouragement, as an interpersonal facilitator to PA was discussed. In relation, some parents attributed a lack of parental encouragement to not knowing how much PA children should do, and a fear of working children too hard or them becoming too tired.

*‘We should encourage them really but I didn’t know that it had to be sixty minutes a day’.*

*(Mother 2, Asian Bangladeshi group)*

It was commonly believed that parents had insufficient knowledge of how to increase their child’s PA levels. Parents lacked awareness of local facilities available for children to be active, and some ethnic minority parents felt language barriers in the community made it difficult to learn about activities being offered.

*‘So we don’t know where to take them, to the sports centres, unless you can speak excellent English and go there’. (Mother 4, Yemeni group)*

Parental lifestyles were thought to impact on children’s opportunities to be active. Parents often believed their work and/ or responsibilities within the home restricted activity levels, because parents promoted sedentary activities to keep children occupied in order for them to continue with other tasks.

*Mother 3: It’s peace and quiet as you say, yeah, peace and quiet*

*Mother 1: So you can get on with your tidying up*

*Mother 7: Yeah, stick the telly or computer on and they’ll be quiet*

*(White British mothers)*

Nevertheless, informal restrictions were placed on children’s screen watching time.

*Mother 2: Yeah, you can’t change what they like… they like a cartoon… if I don’t want them to look I know that I will just switch it off, I will change*

*Father 1: the channel*

*Mother 2: I will put it on the news cos I know they will not like news*

*(Black African group)*

Interpersonal influences extended to the impact of peers on children’s activity levels. Peers were considered a facilitator to PA, providing children with opportunities and motivation to be active. Notably, the absence of peers was thought to limit children’s PA levels.

*‘My daughter does not do enough activity when she is at home because she is alone. She does not have other children to play with so she is discouraged to play out’. (Mother 2, Black Somali group)*

#### Environmental

Several environmental barriers were cited to child PA, including a lack of local facilities, adverse weather and safety issues related to strangers, heavy traffic and unsafe neighbourhoods.

*Mother 3: My child no, because I don’t, because he’s never played out*

*Mother 2: Yeah*

*Mother 3: Because the area that we live in, it’s not good the area that we live in, it’s not safe*

*(Yemeni group)*

Some parents lacked transport and/ or financial resources for children to participate in activities outside of the home and felt their home environment made it difficult for children to be active.

*‘Because I live in an apartment you see so I don’t have a garden or anything you see, so she doesn’t really run around as much as I would like her to’. (Mother 8, White British group)*

### Influences specific to certain ethnic groups

The majority of ethnic-specific themes that arose related to perceived religious and cultural barriers to PA. There was one example, however, where ethnic culture was seen to have a positive influence on children’s PA. Chinese parents reported to regularly take part in PA as a family due to a cultural requirement to be active. Hence, this cultural ethos promoting PA provided children with positive active role models within the family.

*‘Because our nation requires us to pursue all kinds of development including moral, intellectual and physical aspects’. (Mother 6, Chinese group)*

This positive influence of the Chinese culture was however coupled with cultural barriers. Chinese, Asian Bangladeshi and Yemeni parents identified children’s timetables as a barrier to PA. Parents from these groups considered children’s educational commitments, including homework, faith classes and language lessons relating to ethnic background, to delimit the time they have to be active.

*‘You will understand how to balance and you will make your children have sport, for activities, and have time for study too’. (Mother 3, Chinese group)*

Parental expectations of how children should behave also influenced their PA levels. Mothers from Bangladeshi, Black Somali and Yemeni groups cited traditional gender roles associated to ethnic backgrounds and religious values to impact on the PA levels of children. In particular, Asian Bangladeshi, Black Somali and Yemeni girls had less freedom to participate in activities outside the home and were limited in the type of activities they were able to undertake.

*Mother 6: I think for some girls it might be hard [to meet the PA recommendations] because*

*Mother 1: Yeah playing football and things like that*

*Mother 6: A cultural thing, is like girls not to go out too much*

*(Yemeni group)*

*‘After secondary school no way Muslim girls go and play football, to be honest it’s forbidden, you cannot’. (Mother 1, Asian Bangladeshi group)*

The strong emphasis placed on football participation in England (especially so in the city in which the study took place) was therefore considered to restrict girls’ PA levels, and viewed as a potential threat to traditional cultural values among these groups.

*‘In our home country girls use to play hop scotch, marbles, touch and run but here the environment is against them and football is not our culture’. (Mother 1, Black Somali group)*

A lack of appropriate female-only PA facilities was cited as a barrier to PA by Asian Bangladeshi, Black Somali, and Yemeni Muslim mothers. As a result, Muslim girls were considered to experience greater difficulty in achieving the PA recommendation than boys. The lack of female-only facilities was also believed to restrict parents’ own opportunities to be active role models for their children.

*Mother 5: I mean, you know, I wouldn’t mind taking my daughter you know [swimming], when there’s only girls, only females there, I wouldn’t mind*

*Mother 1: You know, but the pool is mixed*

*(Asian Bangladeshi group)*

Therefore, whilst religion *per se* was not considered a barrier to PA, it was thought to restrict the PA levels of children living in Western society.

## Discussion

This article explored parental views of children’s PA (knowledge, attitudes and perceived influences) across six different ethnic groups (Asian Bangladeshi, Black African, Black Somali, Chinese, White British and Yemeni) living in a socio-economically deprived city in North-West England with a relatively low proportion of ethnic minority residents (when compared with the national average). Common factors were reported by all ethnic groups across intrapersonal, interpersonal and environmental levels. Whilst parents valued PA and were aware of its benefits, they were less aware of PA recommendations and expressed a view that schools provided children with sufficient PA. Barriers to PA included a lack of financial resources, safety concerns, limited accessibility of PA opportunities and adverse weather. Parents also reported challenges of motivating their children to be physically active which was attributed in part to preferences for sedentary activities, and several parents described allowing children to watch TV so they were not interrupted in their household tasks. In addition, specific influences on PA were identified that largely reflected cultural influences associated to ethnic background or religious values. In the most part these cultural influences were seen to act in a negative manner against PA, for example through a competing emphasis on educational attainment (Asian Bangladeshi, Chinese and Yemeni groups) or through cultural restrictions on female PA (Asian Bangladeshi, Black Somali, and Yemeni groups). However Chinese parents spoke also of a positive cultural influence, which was the importance of being active as a family.

Our findings suggest the views of ethnic minority parents living in predominantly White-British cities do not differ markedly from those of parents living in cities with a high ethnic diversity [17]. This could be due in part to the similarity in environments at a neighbourhood level, whereby ethnic minority groups live in ethnically-dense, socio-economically deprived areas, regardless of the city’s overall ethnic distribution. The reported barriers to PA reflect those found in other UK studies of parents living in low-SES areas (e.g., [32, 33]). Given the disparities in children’s PA levels [34] and obesity prevalence [35] between low and high SES groups (whereby low SES groups are less physically active, more sedentary and more overweight), an understanding of the barriers to PA amongst low SES groups is important to inform intervention design. Barriers such as cost and transport may be more predominant in low-SES groups [32], whilst other barriers (e.g., safety concerns) are reported by all groups, although the nature of these may vary with SES. For example, Rawlins and colleagues [33] found that children and parents from higher SES backgrounds were most concerned with road safety, and children and parents from lower SES backgrounds were concerned with gangs, violence and dog attacks (factors that were also raised by our participants). Such findings suggest measurement of barriers should go beyond “surface level” to inform PA promotion interventions for children living in low-SES areas.

Whilst the parents in our study were aware of their potential role as facilitators of PA, they reported challenges in supporting their children’s PA and spoke as though they rarely intervened to try and reduce TV viewing time. In a study of parents from predominantly medium-high SES backgrounds in Belgium, De Lepeleere and colleagues [36] found the strategies parents perceived to be effective for supporting children’s PA were in line with the theoretical predictions of social cognitive theory [37] and self-determination theory [38]. For example, parents spoke of using feedback and reward mechanisms, providing children with rationale and choice, and using strategies such as being active with their child or turning a potentially mundane activity (e.g., walking) into something fun (e.g., playing games or singing songs along the way). Therefore it is recommended that interventions targeted at parents from low-SES groups include information about promoting opportunities to be PA and supporting the development of self-determined motivation for PA in their children [32].

In keeping with findings from the DH’s consumer insight summary [17], we found parents to be aware of the health and psycho-social benefits of PA. On the other hand awareness of PA guidelines was low, as observed in other studies of parents from multiple ethnicities in the UK (Black African, Black Caribbean, Indian, Pakistani, Bangladeshi, White, Other [33]) and the US (Hispanic, Black, White [39]), highlighting the importance of communicating PA guidelines effectively to parents [32]. It is notable that a national social marketing campaign (Change4Life,[40]) was in its early stages when this study was undertaken, and may not yet have had chance to take effect. Data published since has shown that Change4Life has improved knowledge and awareness of PA in parents of mixed ethnicities, but this does not necessarily translate to changes in behaviour [41].

Parents in our study held a belief that children were sufficiently physically active at school therefore they did not always see a need to encourage their children to be active out of school hours. This belief has been reported elsewhere [17] and is concerning given the low levels of PA achieved during school physical education (PE) [42] and school recess [43]. Furthermore, local accelerometry data shows Arabic boys are achieving a mean of only 50 min MVPA per weekday [44] which, although greater than their recorded weekend MVPA (38 min per day), is not sufficient to meet the recommended PA levels [2]. Despite the significance of the primary school environment for providing children with opportunities for PA [45], few children are meeting PA recommendations [5] and there are significant variations by ethnic background [46]. Moreover, PA levels decrease further as children reach adolescence [8]. Our findings suggest parents assume children are sufficiently active at school (when they may not be) and are missing opportunities to promote PA out of school. Therefore emphasis needs to be given to promoting PA to children outside of school hours, particularly during weekends which have been identified as a key time for improving PA levels of low-active children [47].

As well as the general themes already discussed, we observed a number of ethnic-specific barriers to PA. Among Asian Bangladeshi, Chinese and Yemeni parents, time available for PA in childhood was decreased by pressure on children to achieve academically and participate in faith and language classes associated to their ethnic heritage. Emphasis on educational attainment in childhood as a barrier to PA is a recurrent theme in research with particular ethnic minority groups (e.g., Chinese [48], Middle Eastern [11], Bangladeshi, Pakistani and Black African [17] populations) living in Western countries. Given parental concern about education, relating the benefits of PA to improved concentration and cognitive functioning [49, 50] may increase engagement in activities.

Ethnicities where the Muslim faith was dominant (Asian Bangladeshi, Black Somali, Yemeni) cited additional barriers to child PA, particularly for females once they reach puberty (i.e. 10 or 11 years of age). Research with religious scholars and Islamic leaders has highlighted that whilst PA is supported by Islam, exercise recommended by healthcare professionals is associated with showy displays of the body (which is discouraged by Islam), and deemed inappropriate for Muslim women to undertake [51]. In the current study, Asian Bangladeshi, Black Somali and Yemeni mothers considered pervasive types of PA in Western culture (e.g., football) a threat to traditional cultural and religious values for Muslim girls and women. However, adolescent girls themselves may not share this view, since football is one of the activities Somali girls report participating in [16]. Mothers in the current study also reported a lack of appropriate facilities for Muslim girls and a lack of knowledge of how to help children be more active. Therefore practical ideas of how to help children meet the PA recommendations are required that are sensitive to traditional cultural gender roles and religious values.

Although parents have an important role to play in promoting children’s PA [12, 13], the extent of cross-group similarities we observed suggests parental attitudes alone cannot account for differences in PA levels between ethnic groups. There is evidence to suggest a combination of cultural and environmental factors may limit the PA opportunities of ethnic minority children living in Western countries [14]. For example, families may lack the finances to purchase appropriate clothing to be active in adverse weather [15], there may be a lack of accessible PA structures in the low SES neighbourhoods where ethnic minority families live [52] or a lack of female-only facilities for PA pursuits [53]. These barriers may have a significant impact on the PA behaviours of children from ethnic minority groups who experience high levels of income poverty [54].

It is recommended that children’s PA interventions address influences at all levels of the socio-ecological model, and account for religious and cultural factors that present additional barriers to PA. At an *intrapersonal* level, parents could be educated with parenting strategies that will help support children’s self-determined motivation for PA, for example through providing a rationale, positive feedback and taking part in activities with children [36]. At an *interpersonal* level, the focus might be on educating parents about PA recommendations, and the importance of supporting their children to achieve these during evenings and weekends. For example, parents could be signposted to free opportunities such as local parks or active transport [32]. Awareness raising amongst Asian Bangladeshi, Chinese and Yemeni groups might focus on the educational benefits of PA. At an *organisational* level, schools need to increase the opportunities for children to be physically active through PE, during recess and throughout the extended school day [55]. For Muslim girls of secondary school age, it is recommended that female-only PA sessions are provided in appropriate school and community settings, girls are permitted to wear cultural dress for exercise, and girls are offered a choice of activities that are deemed culturally acceptable. Finally, at an *environmental* level, intervention is needed to create PA opportunities in low SES neighbourhoods that are safe and accessible to children from all ethnic backgrounds.

### Limitations

The primary purpose of this study was to explore parental views of children’s PA among a multi-ethnic sample, rather than draw between-group comparisons. It is recognised sample sizes of each ethnic group are small and therefore where disparities have been discussed caution must be taken in drawing conclusions. Whilst saturation was reached in terms of cross-group analyses, only one focus group was conducted with each ethnic group therefore it is possible further ethnic-specific subthemes might have emerged if a larger number of groups were conducted.

Despite attempts to recruit parents of both genders, the majority of our participants were mothers. Therefore it would be insightful to conduct research with fathers and extended family members, particularly in Arabic groups (e.g., Asian Bangladeshi, Black Somali, Yemeni) where a strong patriarchal culture dominates and extended family members play a central role in child upbringing. Parental roles in PA promotion may also differ, as shown in a study of African-American girls who perceived their fathers as co-participants and their mothers as facilitators [56]. Whilst parents with children aged 4 to 16 years were eligible to take part in the study, we did not collect data on individual children and are therefore not able to draw comparisons of parental views according to the age of their child. As noted above however, age was a factor that emerged when discussing girls’ PA in the Muslim groups (Asian Bangladeshi, Black Somali, Yemeni). We used postcode data as a proxy for SES, but acknowledge this may not always be synonymous with education level, nor does it provide any indication of the SES status of participants when they were living in their home countries. Therefore findings must be interpreted within the context of the UK living circumstances of the ethnic minority parents in our sample.

Collecting data in one language and analysing it in another has implications for the validity of results but is recognised as a necessary component of cross-cultural research [28, 57]. To help overcome this concern, the quality of translation, including the translators' linguistic competence and knowledge of cultural issues [58], was given important consideration. To overcome any language difficulties for non-native English speakers, attempts were made to recruit bi-lingual trained researchers who were familiar with the research topic and able to conduct the focus groups in the participants’ native language. However this was possible only for the Chinese and Black Somali groups, therefore the Yemeni and Asian Bangladeshi groups were conducted in English with bilingual support from an interpreter as required. The limitations of using multiple facilitators is acknowledged, for it is possible the facilitator’s characteristics (e.g. ethnicity, status) influenced the participants’ level of ease, understanding and the data that emerged. Similarly, the group dynamics may have been influenced by the fact some parents were already known to each other and others were not. To ensure procedures were standardised as far as possible, all facilitators followed the same interview schedule, the Black Somali and Chinese group facilitators were trained by the lead researcher [JT] and JT was present during both these groups.

## Conclusion

This article explored parental views of children’s PA (knowledge, attitudes and perceived influences) within a multi-ethnic sample living in Liverpool, where there is a low proportion of ethnic minority groups in comparison with populations investigated in previous research [17].

We found many similarities in parental views across ethnic groups, including a lack of awareness of PA recommendations, challenges of supporting children’s PA and a perception that children receive sufficient PA whilst at school. Our findings also shed light on how parental views might act as barriers to children’s PA levels, most notably in those cultures with a strong emphasis on educational attainment (Chinese, Yemeni, Asian Bangladeshi) or with a Muslim faith (Asian Bangladeshi, Black Somali, Yemeni). It is recommended children’s PA interventions address influential factors at all levels of the socio-ecological model, and reflect the cultural and religious needs of different ethnic minority groups.

## Abbreviation

PA: Physical activity

## References

[1] World Health Organization (2010). Global recommendations on physical activity for health.

[2] Department of Health (2011). Start active, stay active: a report on physical activity from the four home countries’ chief medical officers.

[3] Healy GN, Dunstan DW, Salmon J, Cerin E, Shaw JE, Zimmet PZ (2008). Breaks in sedentary time: beneficial associations with metabolic risk. Diabetes Care.

[4] Owen N, Healy GN, Matthews CE, Dunstan DW (2010). Too much sitting: the population health science of sedentary behaviour. Exerc Sport Sci Rev.

[5] Scholes S, Mindell J. Physical activity in children*.* In: Health survey for England 2012, Vol 1, Chapter 3. Health and Social Care Information Centre. 2013. http://www.hscic.gov.uk/catalogue/PUB13218 Accessed 14 August 2015.

[6] Woodfield L, Duncan M, Al-Nakeeb Y, Nevill A, Jenkins C (2002). Sex, ethnicity, and socio-economic differences in Children’s physical activity. Pediatr Exerc Sci.

[7] Eyre EL, Duncan ML. The impact of ethnicity on objectively measured physical activity in children. ISRN Obes. 2013; doi:10.1155/2013/757431. 10.1155/2013/757431 PMC3901979 24555154

[8] Brodersen NH, Steptoe A, Boniface DR, Wardle J, Hillsdon M (2007). Trends in physical activity and sedentary behaviour in adolescence: ethnic and socioeconomic differences. Br J Sports Med.

[9] McLeroy KR, Bibeau D, Steckler A, Glanz K (1988). An ecological perspective on health promotion programs. Health Educ Q.

[10] Davison KK, Birch LL (2001). Childhood overweight: a contextual model and recommendations for future research. Obes Rev.

[11] Dwyer GM, Higgs J, Hardy LL, and Baur LA. What do parents and preschool staff tell us about young children's physical activity: a qualitative study. Int J Behav Nutr Phys Act. 2008; doi:10.1186/1479-5868-5-66. 10.1186/1479-5868-5-66 PMC2634757 19077255

[12] Adkins S, Sherwood NE, Story M, Davis M (2004). Physical activity among African-American girls: the role of parents and the home environment. Obes Res.

[13] Cleland V, Venn A, Fryer J, Dwyer T, Blizzard L. Parental exercise is associated with Australian children's extracurricular sports participation and cardiorespiratory fitness: A cross-sectional study. Int J Behav Nutr Phys Act. 2005; doi:10.1186/1479-5868-2-3. 10.1186/1479-5868-2-3 PMC1087868 15811190

[14] Kumanyika SK (2008). Environmental influences on childhood obesity: ethnic and cultural influences in context. Physiol Behav.

[15] Rothe E, Holt C, Kuhn C, McAteer T, Askari I, O’Meara M (2010). Barriers to outdoor physical activity in wintertime among Somali youth. J Immigr Minor Health.

[16] Brophy S, Crowley A, Mistry R, Hill R, Choudhury S, Thomas, NE, et al. Recommendations to improve physical activity among teenagers - A qualitative study with ethnic minority and European teenagers. BMC Public Health. 2011; doi:10.1186/1471-2458-11-412. 10.1186/1471-2458-11-412 PMC3134428 21627781

[17] Department of Health (2008). Healthy weight, healthy lives: consumer insight summary.

[18] Marmot M (2010). Fair society, health society.

[19] Popay J, Williams G (1996). Public health research and Lay knowledge. Soc Sci Med.

[20] Springett J, Owens C, Harrison J (2007). The challenge of combining evidence based practice with "lay" knowledge in health promotion: Fag ends smoking cessation service. Crit Public Health.

[21] Department for Communities and Local Government (2011). The english indices of deprivation 2010.

[22] Office for National Statistics (2011). Population estimates by ethnic group Mid-2009 (experimental) - current estimates.

[23] Trigwell J, Watson PM, Murphy RC, Cable NT, Stratton G (2011). Addressing childhood obesity in black and racial minority (BRM) populations in Liverpool.

[24] Medical Research Council (2008). Developing and evaluating complex interventions: new guidance.

[25] Clark JP, Godlee F, Jefferson T (2003). How to peer review a qualitative manuscript. Peer review in health sciences.

[26] Trigwell J, Watson PM, Murphy RC, Cable NT, Stratton G (2014). Ethnic differences in parental attitudes and beliefs about being overweight in childhood. Health Educ J.

[27] Flick U (2002). An introduction to qualitative research.

[28] Behling O (2000). Translating questionnaires and other research instruments: problems and solutions.

[29] Shenton AK (2004). Strategies for ensuring trustworthiness in qualitative research projects. Educ Inf.

[30] Silverman D (2002). Doing qualitative research: a practical handbook.

[31] Marshall C, Rossman GB (2006). Designing qualitative research.

[32] Bentley GF, Goodred JK, Jago R, Sebire SJ, Lucas PJ, Fox KR (2012). Parents’ views on child physical activity and their implications for physical activity parenting interventions: a qualitative study. BMC Pediatr.

[33] Rawlins E, Baker G, Maynard M, Harding S. Perceptions of healthy eating and physical activity in an ethnically diverse sample of young children and their parents: the DEAL prevention of obesity study. J Hum Nutr Diet. 2012; doi: 10.1111/j.1365-277X.2012.01280. 10.1111/j.1365-277X.2012.01280.x PMC3618369 22827466

[34] Drenowatz C, Eisenmann JC, Pfeiffer KA, Welk G, Heelan K, Gentile D (2010). Influence of socio-economic status on habitual physical activity and sedentary behaviour in 8-to 11-year old children. BMC Public Health.

[35] Stamatakis E, Wardle J, Cole TJ (2010). Childhood obesity and overweight prevalence trends in England: evidence for growing socio-economic disparities. Int J Obes.

[36] De Lepeleere S, De Smet A, Verloigne M, Cardon G, De Bourdeaudhuij I (2013). What practices do parents perceive as effective or ineffective in promoting a healthy diet, physical activity, and less sitting in children: parent focus groups. BMC Public Health.

[37] Bandura A (1986). Social foundations of thought and action: a social cognitive theory.

[38] Ryan RM, Deci EL (2000). Self-determination theory and the facilitation of intrinsic motivation, social development, and well-being. Am Psychol.

[39] Styles JL, Meier A, Sutherland LA, Campbell MK (2007). Parents’ and caregivers’ concerns about obesity in young children. Fam Community Health.

[40] NHS Change4Life. Available at: www.nhs.uk/change4life.

[41] Croker H, Lucas R, Wardle J. Cluster-randomised trial to evaluate the 'Change for Life' mass media/ social marketing campaign in the UK. BMC Public Health, 2012; doi:10.1186/1471-2458-12-404. 10.1186/1471-2458-12-404 PMC3541256 22672587

[42] Fairclough S, Stratton G (2005). Physical activity levels in middle and high school physical education: a review. Pediatr Exerc Sci.

[43] Ridgers ND, Stratton G, Fairclough SJ (2006). Physical activity levels of children during school playtime. Sports Med.

[44] Refaie K (2013). Patterns of physical activity in Arabic Males: barriers and motivations to adopting healthy lifestyles.

[45] Fairclough SJ, Butcher ZH, Stratton G (2008). Primary school children's health-enhancing physical activity patterns: the school as a significant environment?. Education 3–13.

[46] Owen CG, Nightingale CM, Rudnicka AR, Cook DG, Ekelund U, Whincup PH (2009). Ethnic and gender differences in physical activity levels among 9–10-year-old children of white European, South Asian and African-Caribbean origin: the Child Heart and Health Study in England (CHASE Study). Int J Epidemiol.

[47] Fairclough SJ, Boddy LM, Mackintosh KA, Valencia-Peris A, Ramirez-Rico E (2015). Weekday and weekend sedentary time and physical activity in differentially active children. J Sci Med Sport.

[48] Tudor-Locke C, Ainsworth BE, Adair LS, Du S, Popkin BM (2003). Physical activity and inactivity in Chinese school-aged youth: the China health and nutrition survey. Int J Obes Relat Metab Disord.

[49] Sibley BA, Etnier JL (2003). The relationship between physical activity and cognition in children: a meta-analysis. Pediatr Exerc Sci.

[50] Fedewa AL, Ahn S (2011). The effects of physical activity and physical fitness on children’s achievement and cognitive outcomes: a meta-analysis. Res Q Exerc Sport.

[51] Grace C, Begum R, Subhani S, Kopelman P, Greenhalgh T (2009). Understanding barriers to healthy lifestyles in a Bangladeshi community. J Diabetes Nurs.

[52] Molaodi OR, Harding S, Leyland AH, Ellaway A and Kearns A. Area deprivation, ethnic density and fast food outlets, supermarkets and physical activity structures in England. J Epidemiol Community Health. 2010; doi:10.1136/jech.2010.120477.

[53] Gardner K, Salah S, Leavey C, Porcellato L (2010). The perfect size’: perceptions of and influences on body image and body size in young Somali women living in Liverpool – a qualitative study. Divers Health Care.

[54] Palmer G, Kenway P (2007). Poverty rates among ethnic groups in Great Britain.

[55] Hills AP, Dengel DR, Lubans DR (2015). Supporting public health priorities: recommendations for physical education and physical activity promotion in schools. Prog Cardiovasc Dis.

[56] Thompson VJ, Baranowski T, Cullen KW, Rittenberry L, Baranowski J, Taylor WC (2003). Influences on diet and physical activity among middle-class African American 8- to 10-year-old girls at risk of becoming obese. J Nutr Educ Behav.

[57] Weeks A, Swerissen H, Belfrage J (2007). Issues, challenges and solutions in translating study instruments. Eval Rev.

[58] Birbili M (2000). Social research update 31: translating from one language to another.

